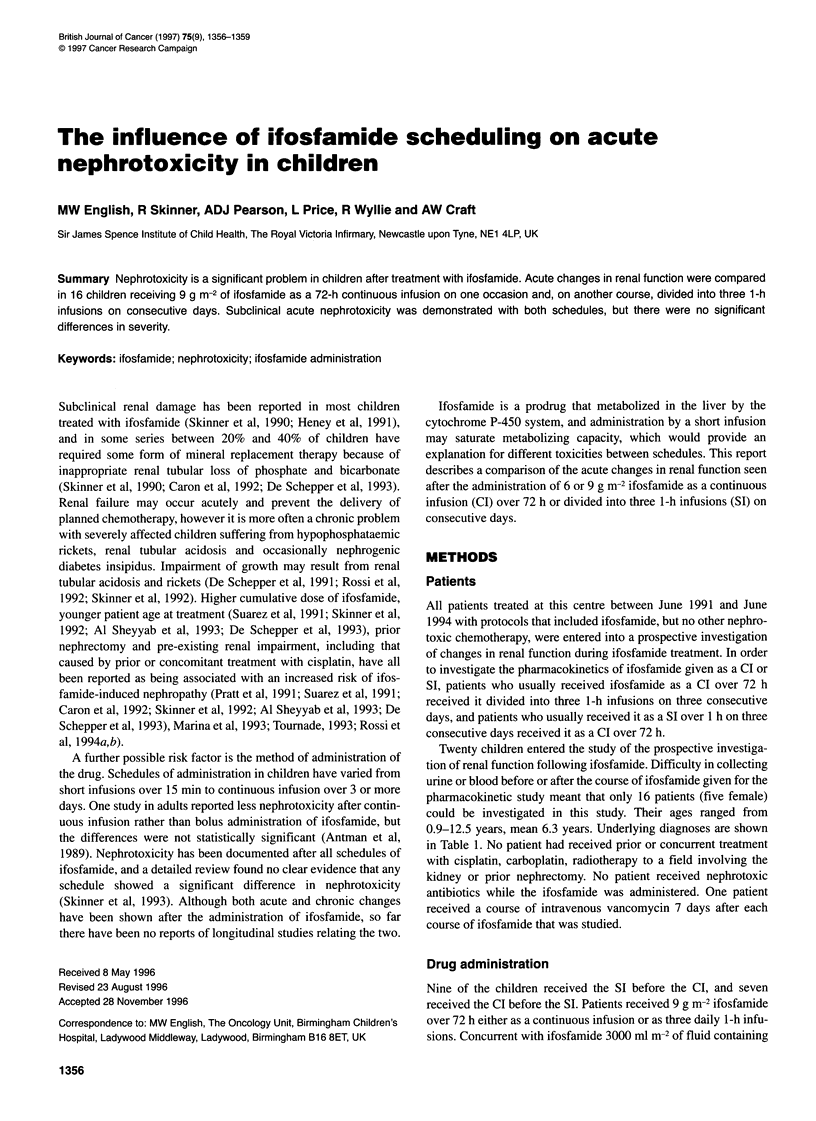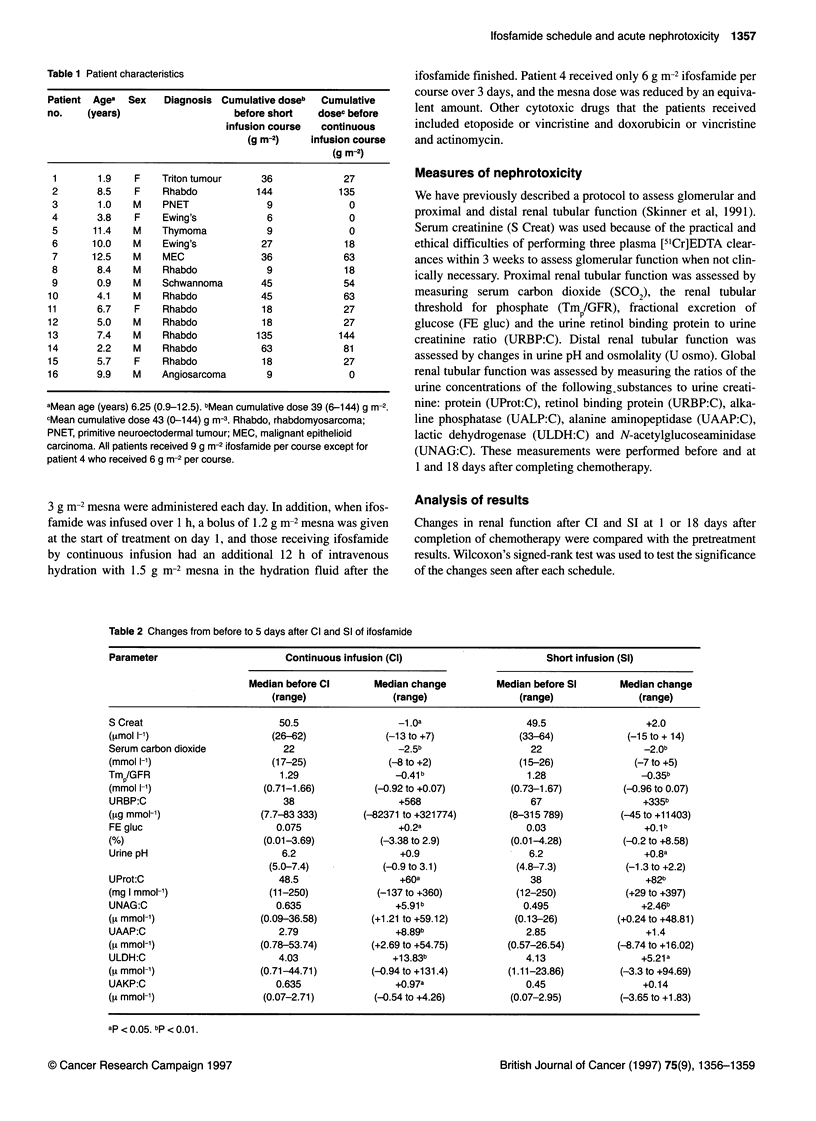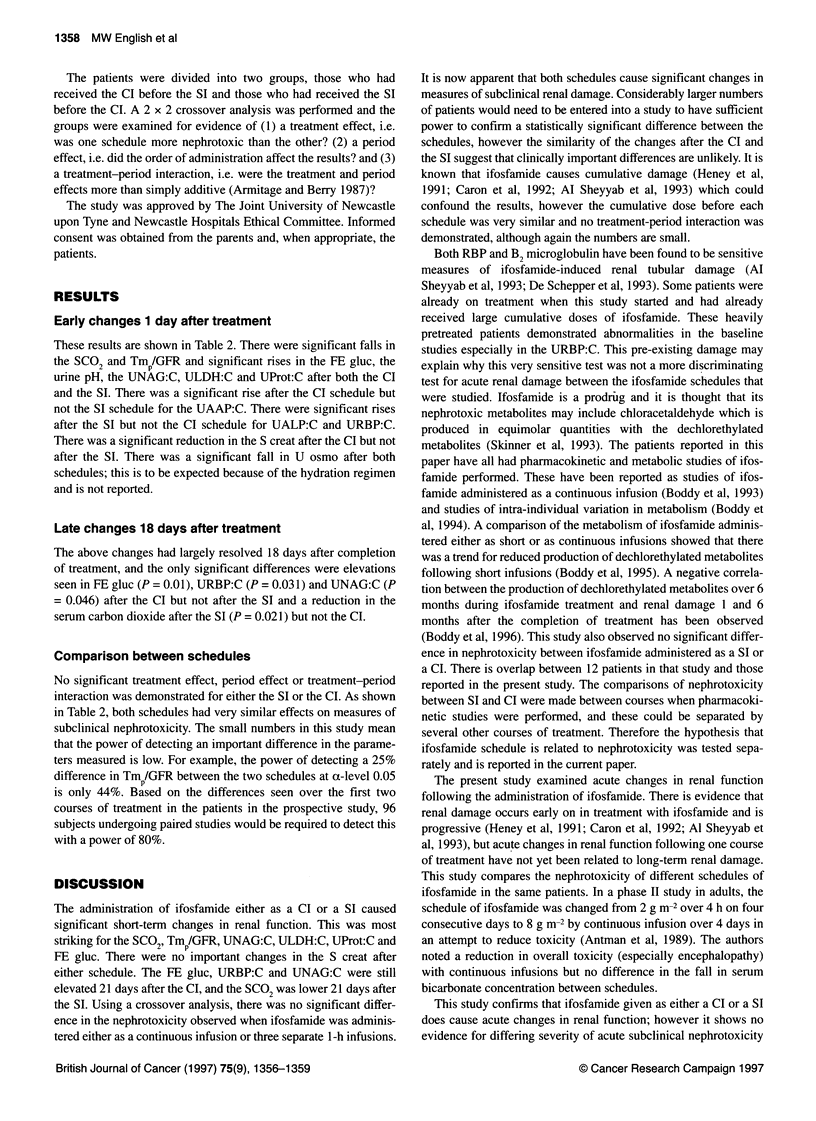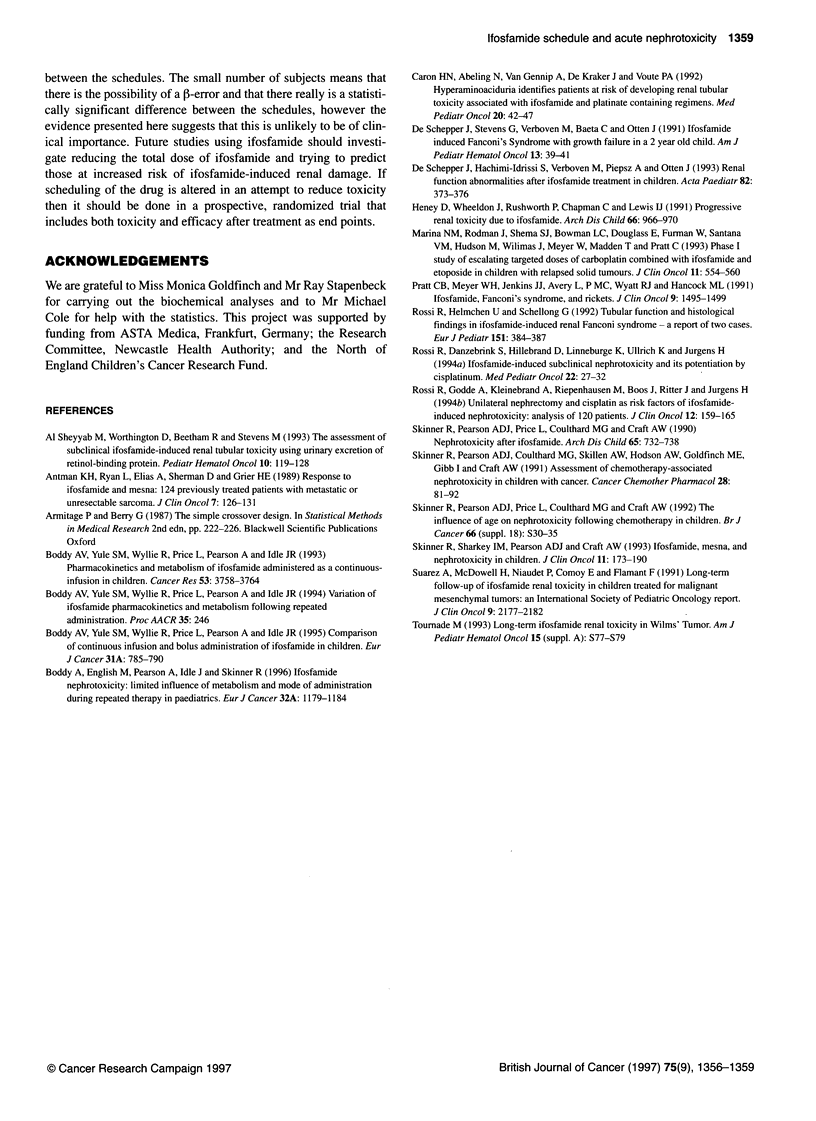# The influence of ifosfamide scheduling on acute nephrotoxicity in children.

**DOI:** 10.1038/bjc.1997.229

**Published:** 1997

**Authors:** M. W. English, R. Skinner, A. D. Pearson, L. Price, R. Wyllie, A. W. Craft

**Affiliations:** Sir James Spence Institute of Child Health, The Royal Victoria Infirmary, Newcastle upon Tyne, UK.

## Abstract

Nephrotoxicity is a significant problem in children after treatment with ifosfamide. Acute changes in renal function were compared in 16 children receiving 9 g m(-2) of ifosfamide as a 72-h continuous infusion on one occasion and, on another course, divided into three 1-h infusions on consecutive days. Subclinical acute nephrotoxicity was demonstrated with both schedules, but there were no significant differences in severity.


British Journal of Cancer (1997) **75**(9), 1356–1359
© 1997 Cancer Research Campaign

### The influence of ifosfamide scheduling on acute nephrotoxicity in children

**MW English, R Skinner, ADJ Pearson, L Price, R Wyllie and AW Craft**

Sir James Spence Institute of Child Health, The Royal Victoria Infirmary, Newcastle upon Tyne, NE1 4LP, UK

**Summary** Nephrotoxicity is a significant problem in children after treatment with ifosfamide. Acute changes in renal function were compared in 16 children receiving 9 g $m^{-2}$ of ifosfamide as a 72-h continuous infusion on one occasion and, on another course, divided into three 1-h infusions on consecutive days. Subclinical acute nephrotoxicity was demonstrated with both schedules, but there were no significant differences in severity.

**Keywords:** ifosfamide; nephrotoxicity; ifosfamide administration

Subclinical renal damage has been reported in most children treated with ifosfamide (Skinner et al, 1990; Heney et al, 1991), and in some series between 20% and 40% of children have required some form of mineral replacement therapy because of inappropriate renal tubular loss of phosphate and bicarbonate (Skinner et al, 1990; Caron et al, 1992; De Schepper et al, 1993). Renal failure may occur acutely and prevent the delivery of planned chemotherapy, however it is more often a chronic problem with severely affected children suffering from hypophosphataemic rickets, renal tubular acidosis and occasionally nephrogenic diabetes insipidus. Impairment of growth may result from renal tubular acidosis and rickets (De Schepper et al, 1991; Rossi et al, 1992; Skinner et al, 1992). Higher cumulative dose of ifosfamide, younger patient age at treatment (Suarez et al, 1991; Skinner et al, 1992; Al Sheyyab et al, 1993; De Schepper et al, 1993), prior nephrectomy and pre-existing renal impairment, including that caused by prior or concomitant treatment with cisplatin, have all been reported as being associated with an increased risk of ifosfamide-induced nephropathy (Pratt et al, 1991; Suarez et al, 1991; Caron et al, 1992; Skinner et al, 1992; Al Sheyyab et al, 1993; De Schepper et al, 1993), Marina et al, 1993; Tournade, 1993; Rossi et al, 1994*a,b*).

A further possible risk factor is the method of administration of the drug. Schedules of administration in children have varied from short infusions over 15 min to continuous infusion over 3 or more days. One study in adults reported less nephrotoxicity after continuous infusion rather than bolus administration of ifosfamide, but the differences were not statistically significant (Antman et al, 1989). Nephrotoxicity has been documented after all schedules of ifosfamide, and a detailed review found no clear evidence that any schedule showed a significant difference in nephrotoxicity (Skinner et al, 1993). Although both acute and chronic changes have been shown after the administration of ifosfamide, so far there have been no reports of longitudinal studies relating the two.

Received 8 May 1996
Revised 23 August 1996
Accepted 28 November 1996

Correspondence to: MW English, The Oncology Unit, Birmingham Children's Hospital, Ladywood Middleway, Ladywood, Birmingham B16 8ET, UK

Ifosfamide is a prodrug that metabolized in the liver by the cytochrome P-450 system, and administration by a short infusion may saturate metabolizing capacity, which would provide an explanation for different toxicities between schedules. This report describes a comparison of the acute changes in renal function seen after the administration of 6 or 9 g $m^{-2}$ ifosfamide as a continuous infusion (CI) over 72 h or divided into three 1-h infusions (SI) on consecutive days.

### METHODS

### Patients

All patients treated at this centre between June 1991 and June 1994 with protocols that included ifosfamide, but no other nephrotoxic chemotherapy, were entered into a prospective investigation of changes in renal function during ifosfamide treatment. In order to investigate the pharmacokinetics of ifosfamide given as a CI or SI, patients who usually received ifosfamide as a CI over 72 h received it divided into three 1-h infusions on three consecutive days, and patients who usually received it as a SI over 1 h on three consecutive days received it as a CI over 72 h.

Twenty children entered the study of the prospective investigation of renal function following ifosfamide. Difficulty in collecting urine or blood before or after the course of ifosfamide given for the pharmacokinetic study meant that only 16 patients (five female) could be investigated in this study. Their ages ranged from 0.9–12.5 years, mean 6.3 years. Underlying diagnoses are shown in Table 1. No patient had received prior or concurrent treatment with cisplatin, carboplatin, radiotherapy to a field involving the kidney or prior nephrectomy. No patient received nephrotoxic antibiotics while the ifosfamide was administered. One patient received a course of intravenous vancomycin 7 days after each course of ifosfamide that was studied.

### Drug administration

Nine of the children received the SI before the CI, and seven received the CI before the SI. Patients received 9 g $m^{-2}$ ifosfamide over 72 h either as a continuous infusion or as three daily 1-h infusions. Concurrent with ifosfamide 3000 ml $m^{-2}$ of fluid containing

**Table 1** Patient characteristics

| Patient no. | Age[a] (years) | Sex | Diagnosis | Cumulative dose[b] before short infusion course (g $m^{-2}$) | Cumulative dose[c] before continuous infusion course (g $m^{-2}$) |
|---|---|---|---|---|---|
| 1 | 1.9 | F | Triton tumour | 36 | 27 |
| 2 | 8.5 | F | Rhabdo | 144 | 135 |
| 3 | 1.0 | M | PNET | 9 | 0 |
| 4 | 3.8 | F | Ewing's | 6 | 0 |
| 5 | 11.4 | M | Thymoma | 9 | 0 |
| 6 | 10.0 | M | Ewing's | 27 | 18 |
| 7 | 12.5 | M | MEC | 36 | 63 |
| 8 | 8.4 | M | Rhabdo | 9 | 18 |
| 9 | 0.9 | M | Schwannoma | 45 | 54 |
| 10 | 4.1 | M | Rhabdo | 45 | 63 |
| 11 | 6.7 | F | Rhabdo | 18 | 27 |
| 12 | 5.0 | M | Rhabdo | 18 | 27 |
| 13 | 7.4 | M | Rhabdo | 135 | 144 |
| 14 | 2.2 | M | Rhabdo | 63 | 81 |
| 15 | 5.7 | F | Rhabdo | 18 | 27 |
| 16 | 9.9 | M | Angiosarcoma | 9 | 0 |

[a]Mean age (years) 6.25 (0.9–12.5). [b]Mean cumulative dose 39 (6–144) g $m^{-2}$. [c]Mean cumulative dose 43 (0–144) g $m^{-3}$. Rhabdo, rhabdomyosarcoma; PNET, primitive neuroectodermal tumour; MEC, malignant epithelioid carcinoma. All patients received 9 g $m^{-2}$ ifosfamide per course except for patient 4 who received 6 g $m^{-2}$ per course.

3 g $m^{-2}$ mesna were administered each day. In addition, when ifosfamide was infused over 1 h, a bolus of 1.2 g $m^{-2}$ mesna was given at the start of treatment on day 1, and those receiving ifosfamide by continuous infusion had an additional 12 h of intravenous hydration with 1.5 g $m^{-2}$ mesna in the hydration fluid after the ifosfamide finished. Patient 4 received only 6 g $m^{-2}$ ifosfamide per course over 3 days, and the mesna dose was reduced by an equivalent amount. Other cytotoxic drugs that the patients received included etoposide or vincristine and doxorubicin or vincristine and actinomycin.

### Measures of nephrotoxicity

We have previously described a protocol to assess glomerular and proximal and distal renal tubular function (Skinner et al, 1991). Serum creatinine (S Creat) was used because of the practical and ethical difficulties of performing three plasma [$^{51}$Cr]EDTA clearances within 3 weeks to assess glomerular function when not clinically necessary. Proximal renal tubular function was assessed by measuring serum carbon dioxide ($SCO_2$), the renal tubular threshold for phosphate ($Tm_p$/GFR), fractional excretion of glucose (FE gluc) and the urine retinol binding protein to urine creatinine ratio (URBP:C). Distal renal tubular function was assessed by changes in urine pH and osmolality (U osmo). Global renal tubular function was assessed by measuring the ratios of the urine concentrations of the following substances to urine creatinine: protein (UProt:C), retinol binding protein (URBP:C), alkaline phosphatase (UALP:C), alanine aminopeptidase (UAAP:C), lactic dehydrogenase (ULDH:C) and *N*-acetylglucoseaminidase (UNAG:C). These measurements were performed before and at 1 and 18 days after completing chemotherapy.

### Analysis of results

Changes in renal function after CI and SI at 1 or 18 days after completion of chemotherapy were compared with the pretreatment results. Wilcoxon's signed-rank test was used to test the significance of the changes seen after each schedule.

**Table 2** Changes from before to 5 days after CI and SI of ifosfamide

| Parameter | Continuous infusion (CI) | | Short infusion (SI) | |
|---|---|---|---|---|
| | Median before CI (range) | Median change (range) | Median before SI (range) | Median change (range) |
| S Creat | 50.5 | −1.0[a] | 49.5 | +2.0 |
| (μmol $l^{-1}$) | (26–62) | (−13 to +7) | (33–64) | (−15 to + 14) |
| Serum carbon dioxide | 22 | −2.5[b] | 22 | −2.0[b] |
| (mmol $l^{-1}$) | (17–25) | (−8 to +2) | (15–26) | (−7 to +5) |
| $Tm_p$/GFR | 1.29 | −0.41[b] | 1.28 | −0.35[b] |
| (mmol $l^{-1}$) | (0.71–1.66) | (−0.92 to +0.07) | (0.73–1.67) | (−0.96 to 0.07) |
| URBP:C | 38 | +568 | 67 | +335[b] |
| (μg $mmol^{-1}$) | (7.7–83 333) | (−82371 to +321774) | (8–315 789) | (−45 to +11403) |
| FE gluc | 0.075 | +0.2[a] | 0.03 | +0.1[b] |
| (%) | (0.01–3.69) | (−3.38 to 2.9) | (0.01–4.28) | (−0.2 to +8.58) |
| Urine pH | 6.2 | +0.9 | 6.2 | +0.8[a] |
| | (5.0–7.4) | (−0.9 to 3.1) | (4.8–7.3) | (−1.3 to +2.2) |
| UProt:C | 48.5 | +60[a] | 38 | +82[b] |
| (mg l $mmol^{-1}$) | (11–250) | (−137 to +360) | (12–250) | (+29 to +397) |
| UNAG:C | 0.635 | +5.91[b] | 0.495 | +2.46[b] |
| (μ $mmol^{-1}$) | (0.09–36.58) | (+1.21 to +59.12) | (0.13–26) | (+0.24 to +48.81) |
| UAAP:C | 2.79 | +8.89[b] | 2.85 | +1.4 |
| (μ $mmol^{-1}$) | (0.78–53.74) | (+2.69 to +54.75) | (0.57–26.54) | (−8.74 to +16.02) |
| ULDH:C | 4.03 | +13.83[b] | 4.13 | +5.21[a] |
| (μ $mmol^{-1}$) | (0.71–44.71) | (−0.94 to +131.4) | (1.11–23.86) | (−3.3 to +94.69) |
| UAKP:C | 0.635 | +0.97[a] | 0.45 | +0.14 |
| (μ $mmol^{-1}$) | (0.07–2.71) | (−0.54 to +4.26) | (0.07–2.95) | (−3.65 to +1.83) |

[a]$P < 0.05$. [b]$P < 0.01$.

© Cancer Research Campaign 1997

The patients were divided into two groups, those who had received the CI before the SI and those who had received the SI before the CI. A 2 × 2 crossover analysis was performed and the groups were examined for evidence of (1) a treatment effect, i.e. was one schedule more nephrotoxic than the other? (2) a period effect, i.e. did the order of administration affect the results? and (3) a treatment–period interaction, i.e. were the treatment and period effects more than simply additive (Armitage and Berry 1987)?

The study was approved by The Joint University of Newcastle upon Tyne and Newcastle Hospitals Ethical Committee. Informed consent was obtained from the parents and, when appropriate, the patients.

### RESULTS

### Early changes 1 day after treatment

These results are shown in Table 2. There were significant falls in the $SCO_2$ and $Tm_p$/GFR and significant rises in the FE gluc, the urine pH, the UNAG:C, ULDH:C and UProt:C after both the CI and the SI. There was a significant rise after the CI schedule but not the SI schedule for the UAAP:C. There were significant rises after the SI but not the CI schedule for UALP:C and URBP:C. There was a significant reduction in the S creat after the CI but not after the SI. There was a significant fall in U osmo after both schedules; this is to be expected because of the hydration regimen and is not reported.

### Late changes 18 days after treatment

The above changes had largely resolved 18 days after completion of treatment, and the only significant differences were elevations seen in FE gluc ($P = 0.01$), URBP:C ($P = 0.031$) and UNAG:C ($P = 0.046$) after the CI but not after the SI and a reduction in the serum carbon dioxide after the SI ($P = 0.021$) but not the CI.

### Comparison between schedules

No significant treatment effect, period effect or treatment–period interaction was demonstrated for either the SI or the CI. As shown in Table 2, both schedules had very similar effects on measures of subclinical nephrotoxicity. The small numbers in this study mean that the power of detecting an important difference in the parameters measured is low. For example, the power of detecting a 25% difference in $Tm_p$/GFR between the two schedules at α-level 0.05 is only 44%. Based on the differences seen over the first two courses of treatment in the patients in the prospective study, 96 subjects undergoing paired studies would be required to detect this with a power of 80%.

### DISCUSSION

The administration of ifosfamide either as a CI or a SI caused significant short-term changes in renal function. This was most striking for the $SCO_2$, $Tm_p$/GFR, UNAG:C, ULDH:C, UProt:C and FE gluc. There were no important changes in the S creat after either schedule. The FE gluc, URBP:C and UNAG:C were still elevated 21 days after the CI, and the $SCO_2$ was lower 21 days after the SI. Using a crossover analysis, there was no significant difference in the nephrotoxicity observed when ifosfamide was administered either as a continuous infusion or three separate 1-h infusions. It is now apparent that both schedules cause significant changes in measures of subclinical renal damage. Considerably larger numbers of patients would need to be entered into a study to have sufficient power to confirm a statistically significant difference between the schedules, however the similarity of the changes after the CI and the SI suggest that clinically important differences are unlikely. It is known that ifosfamide causes cumulative damage (Heney et al, 1991; Caron et al, 1992; Al Sheyyab et al, 1993) which could confound the results, however the cumulative dose before each schedule was very similar and no treatment-period interaction was demonstrated, although again the numbers are small.

Both RBP and $B_2$ microglobulin have been found to be sensitive measures of ifosfamide-induced renal tubular damage (Al Sheyyab et al, 1993; De Schepper et al, 1993). Some patients were already on treatment when this study started and had already received large cumulative doses of ifosfamide. These heavily pretreated patients demonstrated abnormalities in the baseline studies especially in the URBP:C. This pre-existing damage may explain why this very sensitive test was not a more discriminating test for acute renal damage between the ifosfamide schedules that were studied. Ifosfamide is a prodrug and it is thought that its nephrotoxic metabolites may include chloracetaldehyde which is produced in equimolar quantities with the dechlorethylated metabolites (Skinner et al, 1993). The patients reported in this paper have all had pharmacokinetic and metabolic studies of ifosfamide performed. These have been reported as studies of ifosfamide administered as a continuous infusion (Boddy et al, 1993) and studies of intra-individual variation in metabolism (Boddy et al, 1994). A comparison of the metabolism of ifosfamide administered either as short or as continuous infusions showed that there was a trend for reduced production of dechlorethylated metabolites following short infusions (Boddy et al, 1995). A negative correlation between the production of dechlorethylated metabolites over 6 months during ifosfamide treatment and renal damage 1 and 6 months after the completion of treatment has been observed (Boddy et al, 1996). This study also observed no significant difference in nephrotoxicity between ifosfamide administered as a SI or a CI. There is overlap between 12 patients in that study and those reported in the present study. The comparisons of nephrotoxicity between SI and CI were made between courses when pharmacokinetic studies were performed, and these could be separated by several other courses of treatment. Therefore the hypothesis that ifosfamide schedule is related to nephrotoxicity was tested separately and is reported in the current paper.

The present study examined acute changes in renal function following the administration of ifosfamide. There is evidence that renal damage occurs early on in treatment with ifosfamide and is progressive (Heney et al, 1991; Caron et al, 1992; Al Sheyyab et al, 1993), but acute changes in renal function following one course of treatment have not yet been related to long-term renal damage. This study compares the nephrotoxicity of different schedules of ifosfamide in the same patients. In a phase II study in adults, the schedule of ifosfamide was changed from 2 g $m^{-2}$ over 4 h on four consecutive days to 8 g $m^{-2}$ by continuous infusion over 4 days in an attempt to reduce toxicity (Antman et al, 1989). The authors noted a reduction in overall toxicity (especially encephalopathy) with continuous infusions but no difference in the fall in serum bicarbonate concentration between schedules.

This study confirms that ifosfamide given as either a CI or a SI does cause acute changes in renal function; however it shows no evidence for differing severity of acute subclinical nephrotoxicity

© Cancer Research Campaign 1997

between the schedules. The small number of subjects means that there is the possibility of a β-error and that there really is a statistically significant difference between the schedules, however the evidence presented here suggests that this is unlikely to be of clinical importance. Future studies using ifosfamide should investigate reducing the total dose of ifosfamide and trying to predict those at increased risk of ifosfamide-induced renal damage. If scheduling of the drug is altered in an attempt to reduce toxicity then it should be done in a prospective, randomized trial that includes both toxicity and efficacy after treatment as end points.

### ACKNOWLEDGEMENTS

We are grateful to Miss Monica Goldfinch and Mr Ray Stapenbeck for carrying out the biochemical analyses and to Mr Michael Cole for help with the statistics. This project was supported by funding from ASTA Medica, Frankfurt, Germany; the Research Committee, Newcastle Health Authority; and the North of England Children's Cancer Research Fund.

### REFERENCES

Al Sheyyab M, Worthington D, Beetham R and Stevens M (1993) The assessment of subclinical ifosfamide-induced renal tubular toxicity using urinary excretion of retinol-binding protein. *Pediatr Hematol Oncol* **10**: 119–128

Antman KH, Ryan L, Elias A, Sherman D and Grier HE (1989) Response to ifosfamide and mesna: 124 previously treated patients with metastatic or unresectable sarcoma. *J Clin Oncol* **7**: 126–131

Armitage P and Berry G (1987) The simple crossover design. In *Statistical Methods in Medical Research* 2nd edn, pp. 222–226. Blackwell Scientific Publications Oxford

Boddy AV, Yule SM, Wyllie R, Price L, Pearson A and Idle JR (1993) Pharmacokinetics and metabolism of ifosfamide administered as a continuous-infusion in children. *Cancer Res* **53**: 3758–3764

Boddy AV, Yule SM, Wyllie R, Price L, Pearson A and Idle JR (1994) Variation of ifosfamide pharmacokinetics and metabolism following repeated administration. *Proc AACR* **35**: 246

Boddy AV, Yule SM, Wyllie R, Price L, Pearson A and Idle JR (1995) Comparison of continuous infusion and bolus administration of ifosfamide in children. *Eur J Cancer* **31A**: 785–790

Boddy A, English M, Pearson A, Idle J and Skinner R (1996) Ifosfamide nephrotoxicity: limited influence of metabolism and mode of administration during repeated therapy in paediatrics. *Eur J Cancer* **32A**: 1179–1184

Caron HN, Abeling N, Van Gennip A, De Kraker J and Voute PA (1992) Hyperaminoaciduria identifies patients at risk of developing renal tubular toxicity associated with ifosfamide and platinate containing regimens. *Med Pediatr Oncol* **20**: 42–47

De Schepper J, Stevens G, Verboven M, Baeta C and Otten J (1991) Ifosfamide induced Fanconi's Syndrome with growth failure in a 2 year old child. *Am J Pediatr Hematol Oncol* **13**: 39–41

De Schepper J, Hachimi-Idrissi S, Verboven M, Piepsz A and Otten J (1993) Renal function abnormalities after ifosfamide treatment in children. *Acta Paediatr* **82**: 373–376

Heney D, Wheeldon J, Rushworth P, Chapman C and Lewis IJ (1991) Progressive renal toxicity due to ifosfamide. *Arch Dis Child* **66**: 966–970

Marina NM, Rodman J, Shema SJ, Bowman LC, Douglass E, Furman W, Santana VM, Hudson M, Wilimas J, Meyer W, Madden T and Pratt C (1993) Phase I study of escalating targeted doses of carboplatin combined with ifosfamide and etoposide in children with relapsed solid tumours. *J Clin Oncol* **11**: 554–560

Pratt CB, Meyer WH, Jenkins JJ, Avery L, P MC, Wyatt RJ and Hancock ML (1991) Ifosfamide, Fanconi's syndrome, and rickets. *J Clin Oncol* **9**: 1495–1499

Rossi R, Helmchen U and Schellong G (1992) Tubular function and histological findings in ifosfamide-induced renal Fanconi syndrome – a report of two cases. *Eur J Pediatr* **151**: 384–387

Rossi R, Danzebrink S, Hillebrand D, Linneburge K, Ullrich K and Jurgens H (1994*a*) Ifosfamide-induced subclinical nephrotoxicity and its potentiation by cisplatinum. *Med Pediatr Oncol* **22**: 27–32

Rossi R, Godde A, Kleinebrand A, Riepenhausen M, Boos J, Ritter J and Jurgens H (1994*b*) Unilateral nephrectomy and cisplatin as risk factors of ifosfamide-induced nephrotoxicity: analysis of 120 patients. *J Clin Oncol* **12**: 159–165

Skinner R, Pearson ADJ, Price L, Coulthard MG and Craft AW (1990) Nephrotoxicity after ifosfamide. *Arch Dis Child* **65**: 732–738

Skinner R, Pearson ADJ, Coulthard MG, Skillen AW, Hodson AW, Goldfinch ME, Gibb I and Craft AW (1991) Assessment of chemotherapy-associated nephrotoxicity in children with cancer. *Cancer Chemother Pharmacol* **28**: 81–92

Skinner R, Pearson ADJ, Price L, Coulthard MG and Craft AW (1992) The influence of age on nephrotoxicity following chemotherapy in children. *Br J Cancer* **66** (suppl. 18): S30–35

Skinner R, Sharkey IM, Pearson ADJ and Craft AW (1993) Ifosfamide, mesna, and nephrotoxicity in children. *J Clin Oncol* **11**: 173–190

Suarez A, McDowell H, Niaudet P, Comoy E and Flamant F (1991) Long-term follow-up of ifosfamide renal toxicity in children treated for malignant mesenchymal tumors: an International Society of Pediatric Oncology report. *J Clin Oncol* **9**: 2177–2182

Tournade M (1993) Long-term ifosfamide renal toxicity in Wilms' Tumor. *Am J Pediatr Hematol Oncol* **15** (suppl. A): S77–S79

© Cancer Research Campaign 1997